# Minimum 5-Year Follow-up After Arthroscopic Latarjet: Outcomes and Complications

**DOI:** 10.1177/03635465261475810

**Published:** 2026-08-18

**Authors:** Tea Berge, Andrew M. Garratt, Gilbert Moatshe, Anna Grimsmo Øines, Ingvild Blich, Ragnhild Støen, Tom Clement Ludvigsen, Filip Vuletić, Berte Bøe

**Affiliations:** † Oslo University Hospital, Oslo, Norway; ‡ University of Oslo, Oslo, Norway; § Oslo Sports Trauma Research Center, Norwegian School of Sports Center, Oslo, Norway; ‖ Division for Health Services, Norwegian Institute of Public Health, Oslo, Norway; ¶ Stockholm Sports Trauma Research Center, Karolinska Institutet, Stockholm, Sweden; Investigation performed at Oslo University Hospital, Oslo, Norway

**Keywords:** Latarjet procedure, shoulder instability, arthroscopic Latarjet, recurrent anterior shoulder dislocation

## Abstract

**Background:**

The Latarjet procedure is widely used for recurrent anterior shoulder instability with glenoid bone loss. Arthroscopic techniques are increasing; however, there is limited evidence for mid- to long-term outcomes.

**Purpose:**

To evaluate clinical outcomes, recurrence rates, and quality of life at a minimum of 5 years’ follow-up after undergoing the arthroscopic Latarjet procedure.

**Study Design:**

Cohort study; Level of evidence, 3.

**Methods:**

Patients who underwent the arthroscopic Latarjet procedure using double-screw fixation at Oslo University Hospital between 2014 and 2019 were prospectively registered. Descriptive data and Western Ontario Shoulder Instability Index (WOSI) scores were recorded preoperatively and at 1 and 5 years postoperatively. At 5 years, complications and health-related quality of life (HRQoL; EQ-5D-5L) were assessed.

**Results:**

Of 107 patients who underwent the arthroscopic Latarjet procedure, 93 cases met the inclusion criteria. After loss to follow-up, 85 cases were included. The median follow-up time was 5.2 years (range, 5.1-9.4 years). The median age at surgery was 27.8 years (range, 15.6-49.8 years), and 78 of 85 (92%) cases were male. Of the 85 cases, 56 (66%) had >10 dislocations before surgery, and 36 (42%) were revision procedures. At the 5-year follow-up, 2 of 85 patients experienced recurrent dislocations, and 8 of 85 reported subluxations. The median WOSI score improved significantly from 51% (IQR, 29.1%-62.4%) preoperatively to 79% (IQR, 62.9%-91.4%) at 5 years (*P* < .001). The median WOSI score at 1 year was 80% (IQR, 67%-88.4%), with no significant difference between the 1-year and 5-year scores (*P* = .69). The mean EQ-5D-5L index scores at 5 years were 0.889 ± 0.169 compared to 0.896 ± 0.177 for general population norms, with no significant difference (*P* = .77). Overall, 87% of patients reported satisfaction with the surgical outcome. Ten patients underwent reoperation due to complications.

**Conclusion:**

At a minimum 5-year follow-up, the arthroscopic Latarjet procedure resulted in significant improvement in WOSI score, low rates of recurrent dislocation, and high patient satisfaction. The WOSI score remained stable over 1 to 5 years. HRQoL, assessed using the EQ-5D-5L index, was comparable to age- and sex-matched general population norms at 5 years, supporting a satisfactory long-term impact on HRQoL.

Anterior shoulder instability presents a complex challenge in orthopaedic surgery, often requiring surgical intervention to restore joint stability and function. Anterior shoulder dislocation most commonly affects young, active males.^10,34^ Despite both nonoperative and surgical treatment options, some patients experience recurrent instability, pain, and functional impairment, which may limit their participation in work, sports, and recreational activities.^16,17^ Given the young age of this population, the long-term consequences, including health-related quality of life (HRQoL), are of importance.

In cases of recurrent instability, surgical treatment is generally recommended, as either a soft tissue procedure, such as a Bankart repair, or a bony procedure, such as the Latarjet procedure.^19,33,46^ Several risk factors for recurrence after Bankart repair have been identified,^42^ and the recurrence rate varies substantially in the literature. Long-term studies have reported recurrence rates ranging from 18% to 54%, with higher rates observed in younger patients and those with identifiable risk factors.^3,4,11,42^

In Europe, the Latarjet procedure is considered the gold standard surgical procedure for recurrent shoulder instability with significant glenoid bone loss and bipolar bone loss, or after failed previous stabilization surgery.^1^ The procedure can be performed either open or arthroscopically. The open procedure effectively prevents recurrence, although concerns have been raised regarding complication rates.^14,45^

Since its inception, the Latarjet procedure has evolved from open surgery to less invasive approaches, leading to the development of the arthroscopic technique, first described by Lafosse et al^32^ in 2007. The purported advantages of an arthroscopic approach include improved graft positioning, the ability to treat concomitant intra-articular pathologies, less postoperative pain, faster recovery, and fewer complications.^22,31,52^ However, evidence is lacking for improved outcomes compared with the open Latarjet procedure.^15,22,38^ The arthroscopic technique has been increasingly used in clinical practice, and in 2014, Oslo University Hospital moved from open to arthroscopic techniques. The study cohort therefore includes patients treated during the initial years after this transition. The arthroscopic procedure is technically demanding with a learning curve,^6,28^ and surgical experience is associated with shorter operating time and fewer complications.^2^ Evidence for mid- to long-term outcomes after the arthroscopic Latarjet procedure is still lacking, with few published studies, and there is a need for further investigation.^8,9,40,51^

The aim of this study was to evaluate patient-reported shoulder function at a minimum 5-year follow-up after the arthroscopic Latarjet procedure with the Western Ontario Shoulder Instability Index (WOSI) score as the primary outcome. We hypothesized that, with the arthroscopic Latarjet procedure, the preoperative WOSI score would improve at the 5-year follow-up. Secondary outcomes included assessment of generic HRQoL, complications, return to sport, and recurrence rate at the >5-year follow-up.

## Methods

### Ethics and Study Design

The study was conducted in accordance with the Declaration of Helsinki. Approval was obtained from the local Ethics Committee for Medical and Health Research and the Data Protection Office at our institution (protocol No. 20/12294). All participants gave written informed consent. This was a single-center retrospective cohort study.

### Study Population and Surgical Technique

All consecutive patients undergoing the arthroscopic Latarjet procedure with double-screw fixation at our institution between December 2014 and September 2019 were eligible for inclusion. Indications for the Latarjet procedure included a high risk of recurrence, such as that in young male patients; significant glenoid bone loss; bipolar bone loss; a high number of preoperative dislocations, failure after previous soft tissue stabilization, or inadequate capsulolabral tissue. We had no specific threshold for bone loss, either glenoid or bipolar; this was decided by the surgeon for individual patients, and all risk factors for recurrence were evaluated. Patients were included if they had a minimum follow-up of 5 years and had completed primary outcome measures preoperatively and at the 5-year follow-up. Additional inclusion criteria were Norwegian language proficiency and the cognitive and physical ability to complete questionnaires independently.

All procedures were performed by or assisted by the senior author (B.B.) and followed the technique of Lafosse and Boyle.^31^ All patients underwent a standardized arthroscopic procedure, including a diagnostic arthroscopy, preparation of the anterior glenoid neck, harvesting and preparation of the coracoid graft, and graft transfer through a split in the subscapularis tendon with visualization of the axillary and musculocutaneous nerves. The graft was positioned flush with the anteroinferior glenoid rim and secured to the glenoid using two 3.5-mm bicortical screws. The postoperative rehabilitation protocol was the same for all patients. The arm was immobilized in a sling for 4 weeks, with patients allowed to begin range of motion exercises within these limits: flexion and abduction up to 60° and rotation up to 20°, 4 to 6 times daily. After 4 weeks, progression to full range of motion was encouraged. Strengthening exercises were initiated 12 weeks postoperatively, and contact sports were restricted for 4 to 6 months.

### Data Collection

Patients were screened for eligibility based on predefined inclusion criteria, and at a minimum of 5 years postoperatively, they were invited for follow-up and asked to complete an electronic questionnaire that included patient-reported outcome measures (PROMs), recurrence, return to sport, and patient satisfaction. Medical records were reviewed for complications and reoperations.

### Patient-Reported Outcome Measures

The primary outcome was the WOSI score, a validated and widely tested disease-specific PROM^27,44,47,48^ consisting of 21 items that sum to a score of 0 to 100, where higher scores indicate better function and quality of life.^26,27^

For the secondary outcomes, generic HRQoL was assessed using the EQ-5D-5L, which has 5 items or dimensions (mobility, self-care, usual activities, pain/discomfort, and anxiety/depression) with 5 response levels for “health today.” Health states are transformed to a single index using a scoring algorithm derived from valuation tasks usually undertaken with general population samples to give a national value set.^13^ Norwegian EQ-5D index scores range from −0.453 to 1; 1 is the best possible health state, and negative values represent states worse than dead, which is equal to 0.^13^ Included alongside the dimensions, the EQ visual analog scale (VAS) assesses health using a VAS with endpoints labeled “worst imaginable” and “best imaginable” health states. The EQ-5D has evidence for measurement properties in patients undergoing shoulder arthroplasty^36^ and was a secondary outcome in the clinical evaluation of the Latarjet procedure^25,43^ and a primary outcome in the economic evaluation of the procedure.^35,39^

Other patient-completed data included VASs for pain at night, at rest, and during activity. Patient satisfaction, return to sport, and work impact were assessed using yes/no questions. If work was reported to be affected due to shoulder problems, patients were asked to describe their occupation.

Finally, recurrence of instability was assessed using an electronic questionnaire, in which patients were asked whether they had experienced any episodes of shoulder dislocation since surgery (yes/no). Recurrent instability was defined as at least one new glenohumeral dislocation during the follow-up period. Patients were also asked whether they had experienced episodes of subjective instability or subluxation (yes/no), which were analyzed separately from documented dislocations. Complications and reoperations were retrieved from medical records.

### Statistical Analysis

Continuous variables are summarized as mean ± standard deviation or median (interquartile range), dependent on normality as assessed by the Shapiro-Wilk test. Categorical variables are shown as frequency (percent). For comparisons of WOSI scores (preoperative vs 5-year follow-up, and 1-year vs 5-year follow-up), the paired-samples Wilcoxon signed-rank test was used. The results are reported as median (interquartile range) with the corresponding *Z* statistic and *P* value. Response frequencies for the EQ-5D-5L dimensions, EQ-5D-5L index, and EQ-VAS scores were compared with those for Norwegian general population norms for adults >18 years of age^12^ after random exact matching for age and sex. Differences were assessed using the Mann-Whitney *U* test for dimensions and the independent *t* test for the EQ-5D-5L index and EQ-VAS. Statistical significance was set at a *P* value <.05 for all analyses. Missing data were handled pairwise without imputation. Statistical analyses were performed using Stata/SE 18.0 (StataCorp).

## Results

### Study Population

Latarjet surgery was performed on 108 shoulders in 107 patients during the study period. Fifteen patients were not eligible for inclusion because preoperative WOSI scores were unavailable (n = 12), they had an intellectual disability (n = 2), or the patient had died before follow-up (n = 1). Consequently, 93 shoulders met the inclusion criteria and those patients were invited for a 5-year follow-up. Of these, 6 were lost to follow-up and 2 declined participation, leaving 85 shoulders with complete WOSI data preoperatively and at the 5-year follow-up. Four cases had missing WOSI data at 1 year, leaving 81 cases eligible for the preoperative to 1-year and 1-year to 5-year comparisons. The median follow-up time was 5.2 years (range, 5.1-9.4 years).

### Baseline Characteristics

The mean age at surgery was 28.4 ± 7.1 years (range, 15.6-49.8 years), and 78 (92%) cases were male. The operated shoulder was the dominant side in 53 (62%) cases, 36 (42%) cases underwent the arthroscopic Latarjet procedure as a revision procedure after failed previous soft tissue stabilization, and 49 (58%) cases underwent the procedure as primary surgery. Preoperative dislocation burden was substantial, with 56 (66%) cases having experienced >10 dislocations before surgery. An additional procedure, such as remplissage or other concomitant interventions, was performed in 24 (28%) cases. Baseline descriptive and clinical characteristics are summarized in Table 1.

**Table 1:** Baseline Descriptive and Clinical Characteristics (N = 85)*^*a*^*

	Value
Age at surgery, y	
Mean ± SD	28.4 ± 7.1
Median (range)	27.8 (15.6-49.8)
Male sex	78 (92)
Surgical characteristics	
Dominant side operated	53 (62)
Latarjet as revision procedure (after previous stabilization procedure)	36 (42)
Latarjet as primary stabilization procedure	49 (58)
Concomitant procedure performed	24 (28)
Clinical characteristics	
Preoperative dislocations >10	56 (66)
Preoperative dislocations ≤10	29 (34)
Preoperative activity level	
Competitive sport	11 (13)
Recreational sport/exercise	50 (59)

a Data are presented as n (%) unless otherwise indicated.

### Clinical Outcomes

#### Primary Outcome

##### WOSI Score

The median WOSI score improved significantly from 51% (IQR, 29.1%-62.4%) preoperatively to 80% (IQR, 67%-88.4%) at the 1-year follow-up (*P* < .001) and remained stable at 79% (IQR, 62.9%-91.4%) at the 5-year follow-up. The median improvement from baseline to the 5-year follow-up was 29.0 percentage points (IQR, 10.8-38.3), and there was no significant difference in WOSI scores from the 1-year to 5-year follow-up (*P* = .69) (Figure 1).

**Figure 1.**
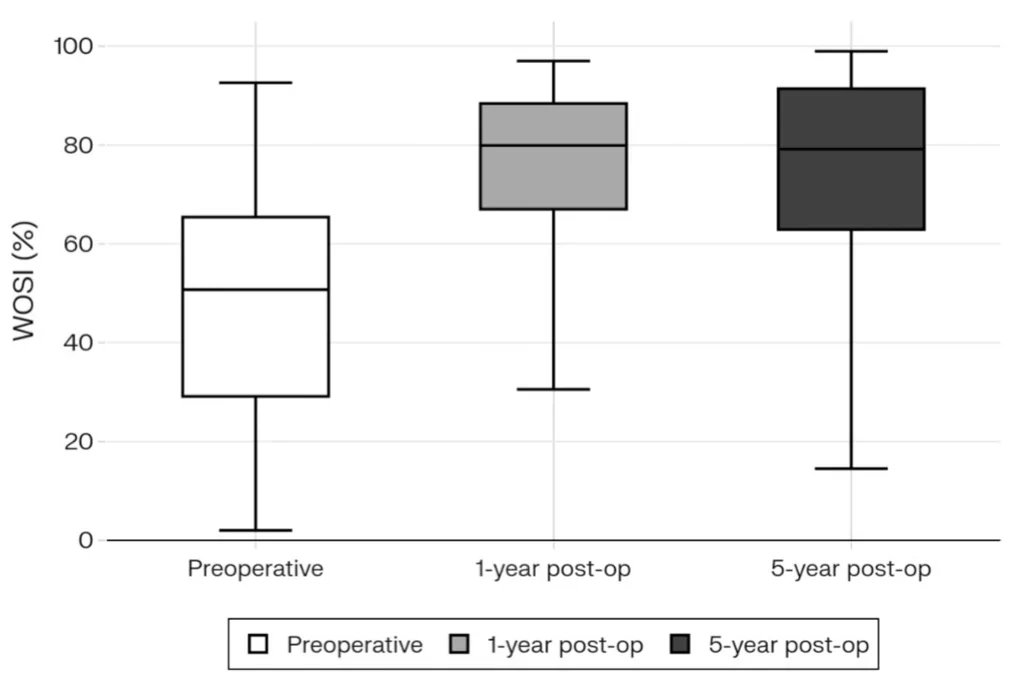
Median Western Ontario Shoulder Instability Index (WOSI) scores (%) preoperatively and at 1 year and 5 years of follow-up. Each box shows the median and interquartile range. Higher values indicate better shoulder function (N = 85).

#### Secondary Outcomes

##### EQ-5D-5L

At the 5-year follow-up, the majority of cases had no problems in mobility (93%), self-care (88%), usual activities (77%), and anxiety/depression (73%). Pain/discomfort was the dimension with the lowest proportion of no problems reported (35%), followed by slight problems in 48% and moderate problems in 14% (Table 2). Figure 2 shows that the distribution of responses was very similar to that of the matched general population norms for mobility, usual activities, and anxiety/depression. When compared to the norms, a greater proportion of patients reported problems with self-care (78% vs 71%; *P* = .048) and pain/discomfort (65% vs 44%; *P* = .002). The mean EQ-5D-5L index scores were 0.889 ± 0.169 and 0.896 ± 0.177 for patients and norms, respectively, which was not significantly different (*P* = .77). The mean EQ-VAS score at the 5-year follow-up was 70.4 ± 23.2, compared with 79.1 ± 16.6 in the general population (*P* = .0065). Furthermore, patient scores spanned the full range of the EQ-VAS (0 to 100), compared with the lowest score of 15 in the general population.

**Table 2:** EQ-5D-5L Frequencies and Descriptive Statistics for General Population and Latarjet Cases (n = 81) at 5 Years*^*a*^*

EQ-5D Dimension/Scores	No Problems	Slight Problems	Moderate Problems	Severe Problems	Unable/Extreme
General population					
Mobility	74 (91.4)	5 (6.2)	00 (0.0)	2 (2.5)	00 (0.0)
Self-care	78 (96.3)	1 (1.2)	2 (2.5)	00 (0.0)	00 (0.0)
Usual activities	68 (84.0)	7 (8.6)	3 (3.7)	00 (0.0)	3 (3.7)
Pain/discomfort	45 (55.6)	32 (39.5)	2 (2.5)	1 (1.2)	1 (1.2)
Anxiety/depression	56 (69.1)	16 (19.8)	3 (3.7)	4 (4.9)	2 (2.5)
Latarjet patients					
Mobility	75 (92.6)	3 (3.7)	2 (2.5)	1 (1.2)	00 (0.0)
Self-care	71 (87.7)	8 (9.9)	1 (1.2)	1 (1.2)	00 (0.0)
Usual activities	62 (76.5)	13 (16.0)	4 (4.9)	2 (2.5)	00 (0.0)
Pain/discomfort	28 (34.6)	39 (48.1)	11 (13.6)	3 (3.7)	00 (0.0)
Anxiety/depression	59 (72.8)	17 (21.0)	2 (2.5)	2 (2.5)	1 (1.2)
	Mean ± SD	Best Possible	Worst Possible	No. of Health States*^*b*^*	Range*^*c*^*
General population					
EQ-5D-5L index	0.896 ± 0.177	34 (42.0)	0.13 (0.0)	21	0.137 to 1
EQ-VAS	79.1 ± 16.6	3 (3.7)	00 (0.0)	23	15 to 100
Latarjet patients*^*d*^*					
EQ-5D-5L index	0.889 ± 0.169	25 (30.9)	−0.126 (0.0)	24	−0.126 to 1
EQ-VAS	70.4 ± 23.2	5 (6.2)	1 (1.2)	31	00 to 100

a Data are presented as n (%) unless otherwise indicated. VAS, visual analog scale.

b Number of possible health states: EQ-5D-5L = 5^5^ (3125), EQ-VAS = 101.

c EQ-5D-5L index scores range from −0.453 to 1, where 1 is the best possible health state; EQ-VAS scores range from 0 to 100, where 100 is the best possible health state.

d A statistically significant difference was found for the EQ-VAS score (*P* = .006), *self-care* (*P* = .048), and pain/discomfort (*P* = .002). No significant differences were found for the remaining EQ-5D-5L dimensions (Mann-Whitney *U* test) or for the EQ-5D-5L index score (*t* test) compared with the general population.

**Figure 2.**
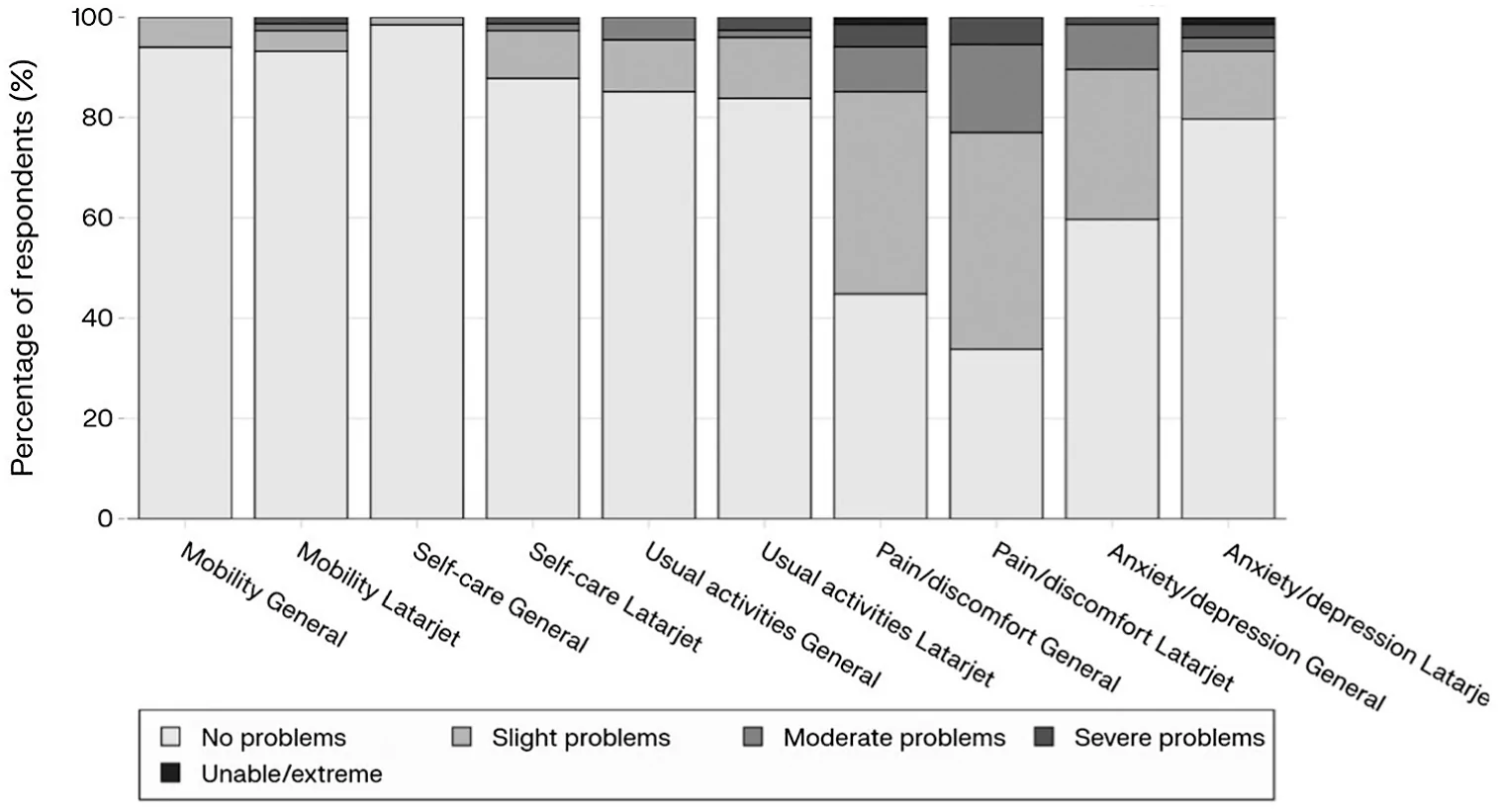
Frequencies for EQ-5D-5L dimensions/scores compared to general population (n = 81).

##### Recurrence of Instability

At the 5-year follow-up, 2 of the 85 (2%) cases experienced recurrent glenohumeral dislocations; 1 underwent revision stabilization surgery. Subjective episodes of instability or subluxation, with no documented dislocations, were reported by 8 (9%) patients; the median number of events was 2 (range, 1-5).

##### Complications and Reoperations

Ten (12%) cases underwent at least one reoperation within 5 years of the index procedure. Indications for reoperation included screw-related problems in 4 (5%) cases, graft failure in 3 (4%) cases, postoperative stiffness requiring surgical intervention in 2 (2%) cases, and deep infection in 1 (1%) case. No permanent neurological complications were recorded in the cohort. Two cases experienced transient musculocutaneous nerve symptoms, both resolved spontaneously. The 3 graft failures were early postoperative events and were managed with open revision surgery within 2 weeks. Two cases involved early graft loosening detected on postoperative computed tomography scans, and 1 case involved early graft fracture with subsequent screw removal and refixation with a suspensory device. One of these patients later experienced recurrent instability and underwent additional procedures during follow-up, including revision stabilization.

Screw removals were performed at 8, 24, 28, and 43 months postoperatively. Arthrolysis procedures were performed within the first year after the index operation, and arthroscopic rinse for suspected deep infection was performed 2 weeks postoperatively.

##### Pain Assessment

Pain levels at the 5-year follow-up were generally low. The median VAS pain scores were 0 (IQR, 0-1.0; range, 0-10) for night pain, 0 (IQR, 0-1.0; range, 0-9) at rest, and 1.0 (IQR, 0-3.0; range, 0-10) during activity. Compared to those not reoperated, patients who underwent reoperation after the primary Latarjet procedure reported higher VAS pain scores at rest (*P* = .017) and during activity (*P* = .048).

##### Return to Sport and Patient Satisfaction

Preoperatively, 61 of 85 (72%) patients reported participation in sport, either at competitive (n = 11) or recreational levels (n = 50). At the 5-year follow-up, 49 (80%) of these patients reported being regularly active in sport. Among the 36 patients who were not active at follow-up, only 7 reported that shoulder-related problems were the reason for discontinuation of sport. However, not all patients returned to their previous activity level or sport type, with several reporting a shift from high-risk contact or overhead sports to lower-demand activities such as fitness training, cycling, or running. Overall, 74 of 85 (87%) patients reported satisfaction with their surgical outcomes (Table 3).

**Table 3:** Patient-Reported and Clinical Outcomes at 5-Year Follow-up (N = 85)*^*a*^*

Outcome	Value
WOSI score	
1 y	80 (67.0-88.4)
5 y	79 (67.0-91.4)
Change preoperative to 5 y	29.0 (10.8-38.3)
*P* value (preop vs 5 y)	<.001
*P* value (1 y vs 5 y)	.69
Pain assessment (VAS score 0-10)	
Night	00 (0-1.0)
Rest	00 (0-1.0)
Activity	1.0 (0-3.0)
EQ-5D-5L score	
EQ-5D-5L	0.889 ± 0.169
EQ-VAS (n = 81)	70.4 ± 23.2
Activity and satisfaction	
Overall satisfaction	74 (87)
Return to any sport (n = 61)	49 (80)
Shoulder affects work	16 (19)
Recurrence and instability	
Recurrent dislocation	2 (2)
Subjective subluxation	8 (9)
Complications and reoperation	
Any reoperation	10 (12)
Screw-related complications	4 (5)
Graft failure/nonunion	3 (4)
Stiffness requiring surgery	2 (2)
Infection	1 (1)

a Data are presented as n (%), median (IQR), or mean ± SD unless otherwise indicated. VAS, visual analog scale; WOSI, Western Ontario Shoulder Instability Index.

##### Work Impact

Among 85 patients who provided work-related data, 16 (19%) reported that the shoulder affected their work capacity or ability to perform specific tasks. The majority (12/16) of these patients had physically demanding occupations, including carpentry (n = 4), construction work (n = 2), health care work involving lifting (n = 2), and manual labor (n = 4). One patient working as a carpenter reported permanent work disability directly attributable to the shoulder condition (Table 3).

## Discussion

The main finding of this study was a significant improvement in the WOSI score from baseline to the 1-year follow-up, which was sustained at 5 years. The median WOSI score improved from 51% preoperatively to 80% at 1 year and remained stable at 79% at 5 years, suggesting that the improvement in function from preoperative levels was maintained over time. The WOSI score observed in this cohort was somewhat lower than those reported in previous studies of both open and arthroscopic Latarjet procedures.^9^ However, comparisons with existing studies reporting midterm outcomes after the arthroscopic Latarjet procedure are limited due to the variation in shoulder-specific outcome measures used, including the Rowe, Walch, Duplay, or Oxford Shoulder Score.^5,8,40,51^ Consequently, direct comparison of outcomes of shoulder function is limited.

Furthermore, there is relatively limited evidence for mid- to long-term outcomes after arthroscopic Latarjet procedures. To our knowledge, only a small number of studies have reported outcomes with a minimum follow-up of 5 years,^8,9,40,51^ highlighting the importance of the present study in providing data for longer-term outcomes. The lower WOSI score observed in our cohort may partly reflect its high-risk profile. In Norway, the indication for a Latarjet procedure includes patients with previously failed soft tissue stabilization, such as a Bankart procedure, as well as patients considered at high risk of recurrence. In this cohort, 42% of the Latarjet procedures were revision cases, 66% of patients had >10 preoperative dislocations, and a substantial proportion had radiologically documented glenoid bone loss, with a median glenoid bone loss of 16% (IQR, 9%-23%). Such patients represent a group with a substantially heavier preoperative burden than most frequently reported. In some countries, most notably France, the Latarjet technique is frequently used as a primary index procedure for shoulder instability.^9,50^ Cohorts with less complex preoperative pathology may be expected to demonstrate superior outcomes.

While there are several purported advantages of the arthroscopic approach compared to open surgery,^22,31,52^ our findings did not support this, including the 12% reoperation rate.

The EQ-5D-5L was not completed by patients preoperatively; however, at the 5-year follow-up EQ-5D-5L index scores were comparable to those for age- and sex-matched general population norms. In the absence of preoperative EQ-5D-5L scores, this might suggest that patients’ general levels of HRQoL returned to levels experienced before shoulder instability. While patients reported more problems with self-care and greater levels of pain/discomfort compared with the general population, no differences were observed for mobility, usual activities, or anxiety/depression. Despite these differences in individual dimensions, the overall EQ-5D-5L index score did not differ significantly between the groups.

EQ-VAS scores were 8.7 points lower than those of the age- and sex-matched general population, which was significant. The EQ-VAS assesses general health, and patients may consider other aspects of health not included in the 5 EQ-5D-5L dimensions, such as sleep or vitality. Moreover, these may relate to ongoing shoulder problems. A small number of patients (n = 2) reported very low EQ-VAS scores, which contributed to the lower mean. However, these patients reported excellent shoulder-specific outcomes, suggesting that low EQ-VAS scores may occur despite satisfactory shoulder function. Minimal important differences for the EQ-VAS have been estimated in the range of 5.35 to 10.00,^18,37,49^ and therefore the observed difference may be clinically meaningful. This finding highlights the importance of the EQ-VAS as a more subjective measure of HRQoL that provides additional important information alongside the index. Inclusion of the EQ-5D-5L preoperatively or as a retrospective preshoulder instability assessment would have enhanced understanding of general HRQoL outcomes in these patients at long-term follow-up. Future research in patients undergoing arthroscopic Latarjet surgery should also assess whether there are longitudinal differences in outcomes assessed by these 2 components of the EQ-5D-5L.

General population data were matched for age and sex. Patients with shoulder instability are often engaged in competitive or recreational sports.^29,30^ The relatively high proportion of patients participating in sport (72%) in this cohort may reflect the selection of patients undergoing Latarjet surgery, who frequently represent a high-risk group for recurrent instability.^3^ Shoulder stabilization surgery has been associated with lower recurrence rates and higher rates of return to sport, both of which are identified as important key factors of patient satisfaction.^20,41,53^

In this physically active patient cohort, residual symptoms of functional limitations may have a greater impact on perceived health status and expectations than in the general population. Although most of the active patients preoperatively reported continued participation in sport at the 5-year follow-up, a substantial proportion did not return to their previous activity level or type of sport. The rate of return to sport (80%) was somewhat lower than that reported in previous studies after both open and arthroscopic Latarjet procedures (85%-94%).^8,23,24,40^

Some patients reported transitioning from high-risk contact or overhead sports to lower-demand activities. Higher expectations regarding physical performance and return to sport may influence perceived health despite overall satisfactory clinical outcomes.

Overall, patient satisfaction after the arthroscopic Latarjet procedure was high, consistent with previous reports.^8,40^ This finding suggests that satisfactory subjective outcomes may be achieved even in the presence of residual symptoms, reduced sport participation, or altered activity levels.

Complications after both open and arthroscopic Latarjet surgery reported in the literature include recurrent instability, graft-related complications, infection, hardware-related problems, stiffness, residual pain, and neurological complications.^21^ In the present study, 8 (9%) patients reported subluxation episodes, although subluxation is a subjective symptom that may be difficult for patients to report accurately. Two (2%) patients experienced a new dislocation during follow-up, one during an epileptic seizure and the other after a shoulder trauma.

Previous studies have reported complication rates as high as 30% after the open Latarjet procedure, with reoperation rates of 7% found in a systematic review.^14^ In the present study, 10 (12%) cases required reoperation. Three of these reoperations were due to early graft failure, and 2 occurred within the first 20 cases after the institution transitioned from an open to an arthroscopic technique.

When transitioning from an open to an arthroscopic approach, a learning curve is expected, during which complication rates tend to decrease after the first 20 to 30 procedures.^2,28^ The relatively lower WOSI scores, higher recurrence rate, and reoperation rate observed in this study may therefore, at least in part, be attributed to the surgeon's learning curve with the arthroscopic technique.^2^

The main limitations of this study are the relatively small sample size and the retrospective design, which lacks a control group for direct comparison. Although some data, such as WOSI score, were collected prospectively, baseline EQ-5D-5L values were absent for direct comparison. The inclusion of other shoulder-specific measures of HRQoL such as the Oxford Shoulder Score^7^ may have contributed to the generalizability of the results but would have increased respondent burden to patients. In addition, loss to follow-up represents a challenge in this relatively young patient population. However, follow-up was obtained for 85 of 93 (91%) eligible cases, which strengthens the validity of the results.

## Conclusion

At a minimum 5-year follow-up, the arthroscopic Latarjet procedure resulted in significant improvement in WOSI score, low rates of recurrent dislocation, and high patient satisfaction. The WOSI score remained stable over 1 to 5 years. HRQoL, assessed by the EQ-5D-5L index and 4 of the 5 contributing dimensions, was comparable to age- and sex-matched general population norms at 5 years, supporting satisfactory long-term outcomes covering important aspects of health. However, EQ-VAS scores were poorer, and the number reporting pain/discomfort was higher compared to the general population at 5 years.
